# In Vitro Activity of Zoliflodacin Against *Neisseria gonorrhoeae* Isolates from Shanghai, China (2020–2023)

**DOI:** 10.3390/antibiotics15010061

**Published:** 2026-01-05

**Authors:** Linxin Yao, Tingli Tian, Xinying Lu, Danyang Zou, Zhuojun Tang, Xin Feng, Tong Zheng, Zhen Ning, Yi Lin, Meiping Ye, Jianping Jiang, Pingyu Zhou

**Affiliations:** 1 STD Institute, Shanghai Skin Disease Hospital, Tongji University School of Medicine, Shanghai 200443, China; 2 Department of Dermatology, Xinhua Hospital, Shanghai Jiao Tong University School of Medicine, Shanghai 200092, China; 3 Division of Tuberculosis and HIV/AIDS Prevention, Shanghai Municipal Center for Disease Control and Prevention, Shanghai 200051, China; 4 Institute of Antibiotics, Huashan Hospital, Fudan University, Shanghai 200040, China

**Keywords:** *Neisseria gonorrhoeae*, zoliflodacin, in vitro activity, QRDR

## Abstract

**Background/Objectives:** The escalating threat of drug-resistant *Neisseria gonorrhoeae* underscores the urgent need for novel therapeutic agents. Zoliflodacin, a first-in-class spiropyrimidinetrione antibiotic that targets bacterial DNA gyrase and topoisomerase IV, represents a promising candidate for gonorrhea treatment. **Methods:** From 2020 to 2023, a total of 876 urogenital *N. gonorrhoeae* isolates were collected from 35 hospitals across Shanghai, China. In vitro susceptibilities to zoliflodacin and six conventional antibiotics (penicillin, tetracycline, ciprofloxacin, azithromycin, ceftriaxone, and spectinomycin) were determined using the agar dilution method. Whole-genome sequencing was conducted to identify sequence types (STs) and amino-acid substitutions in GyrA, GyrB, ParC, ParE, and MtrR. **Results:** Zoliflodacin exhibited potent in vitro activity, with minimum inhibitory concentrations (MICs) ranging from ≤0.004 to 0.25 mg/L (MIC_50_ = 0.06 mg/L; MIC_90_ = 0.125 mg/L), all below the breakpoint (0.5 mg/L). Notably, zoliflodacin maintained high activity against isolates resistant to ceftriaxone, azithromycin, ciprofloxacin, penicillin, and tetracycline. Although all isolates were susceptible to zoliflodacin, elevated MIC values were observed in ST7363 and ST8123 compared with other clones. Genomic analysis identified no substitutions associated with increased zoliflodacin MICs, and most GyrB sequences, the key gene associated with zoliflodacin resistance, remained intact. **Conclusions:** These findings demonstrate that zoliflodacin possesses robust activity against circulating multidrug-resistant *N. gonorrhoeae* lineages in Shanghai and support its potential clinical use for the treatment of gonorrhea. Continued genomic and phenotypic surveillance is warranted to preserve the long-term efficacy of this novel agent.

## 1. Introduction

Gonorrhea poses a significant global public health challenge, with antimicrobial resistance in *Neisseria gonorrhoeae* representing a particular concern [1,2]. *N. gonorrhoeae* has developed resistance to all commonly used antimicrobial classes, including sulfonamides, penicillins, tetracyclines, and fluoroquinolones [3]. Currently, intramuscular ceftriaxone is recommended as the primary treatment for gonorrhea in both China and the United States [4,5], and European guidelines recommend combining it with azithromycin [6]. However, the global emergence and spread of ceftriaxone-resistant *N. gonorrhoeae* underscores the urgent need to develop novel antibacterial drugs to combat this growing threat [7].

The DNA gyrase (composed of GyrA and GyrB) and topoisomerase IV (composed of ParC and ParE) of *N. gonorrhoeae* are essential type II topoisomerases that play crucial roles in promoting DNA replication and transcription [8]. Several novel topoisomerase II inhibitors with potent activity against *N. gonorrhoeae* are currently under development. These include zoliflodacin, gepotidacin, and TP0480066, among others [8,9,10]. Zoliflodacin is a novel spiropyrimidinetrione antibiotic that induces DNA breaks mediated by gyrase and topoisomerase IV. Unlike fluoroquinolones, zoliflodacin primarily interacts with the GyrB subunit [8]. In vitro selection experiments with TP0480066 have identified substitutions in ParE in resistant mutants, although its precise target preference requires further elucidation [10]. In contrast, gepotidacin exhibits a well-balanced dual-targeting activity against both gyrase and topoisomerase IV in *N. gonorrhoeae* [11]. These novel topoisomerase II inhibitors exhibit potent antibacterial activity against *N. gonorrhoeae*, including strains resistant to fluoroquinolones. The phase III clinical trial demonstrated that a single 3-g oral dose of zoliflodacin achieved non-inferiority compared with intramuscular ceftriaxone combined with oral azithromycin [12]. Given zoliflodacin’s promising clinical efficacy and its unbalanced target interaction profile, understanding the pre-existing genetic landscape for potential resistance becomes an urgent public health priority.

Previous studies have shown that zoliflodacin has high activity in vitro against *N. gonorrhoeae* [13,14,15,16,17]. To date, only a single clinical isolate of *N. gonorrhoeae* with reduced susceptibility to zoliflodacin has been reported globally, the H035 strain from Japan, identified in 2000, which carries the GyrB-D429V substitution [18]. In contrast, in vitro selection experiments have identified the GyrB-D429A/N or K450T substitutions as first-step mutations that confer an increase in the minimum inhibitory concentration (MIC) of zoliflodacin (0.5–4 mg/L) [19,20]. These findings highlight the urgent need for large-scale, continuous surveillance of mutations in type II topoisomerases among clinical gonococcal isolates prior to the widespread implementation of zoliflodacin therapy.

In this multicenter study, we collected 876 *N. gonorrhoeae* isolates from 35 hospitals across different districts of Shanghai, China between 2020 and 2023. We assessed the in vitro susceptibility of these isolates to zoliflodacin and compared zoliflodacin MICs between drug-susceptible and drug-resistant strains, as well as among strains belonging to different multilocus sequence typing (MLST) clones. Finally, mutations in genes associated with zoliflodacin resistance, including *gyrA*, *gyrB*, *parC*, *parE*, and *mtrR* were analyzed. Together, our findings provide evidence supporting the potential clinical use of zoliflodacin for the treatment of *N. gonorrhoeae* infections.

## 2. Results

### 2.1. Clinical and Microbiological Characteristics

In this study, we collected 876 *N. gonorrhoeae* isolates (one per patient) from the urogenital tracts of patients attending 35 hospitals across Shanghai, China, between 2020 and 2023. The majority of patients were male (85.73%, 751/876), over 20 years of age (93.49%, 819/876), and primarily presented with urethral infections (86.07%, 754/876) (Table 1). Most isolates (75.91%, 665/876) were obtained from hospitals located in suburban regions, reflecting the widespread distribution of gonorrhea cases across both central and peripheral healthcare facilities in Shanghai.

The susceptibilities of all isolates to six commonly used antimicrobials were evaluated (Table 2). High resistance rates were observed for ciprofloxacin (99.54%, 872/874), tetracycline (78.54%, 688/876), and penicillin (78.20%, 685/876). The non-susceptible rates to ceftriaxone and azithromycin were 6.85% (60/876) and 9.70% (85/876), respectively. All isolates remained susceptible to spectinomycin. These findings suggest the continued effectiveness of spectinomycin, ceftriaxone, and azithromycin in Shanghai, while underscoring the need for ongoing surveillance of emerging ceftriaxone and azithromycin resistance.

### 2.2. In Vitro Activity of Zoliflodacin Against Drug-Susceptible and Drug-Non-Susceptible N. gonorrhoeae Isolates

**Figure 1:**
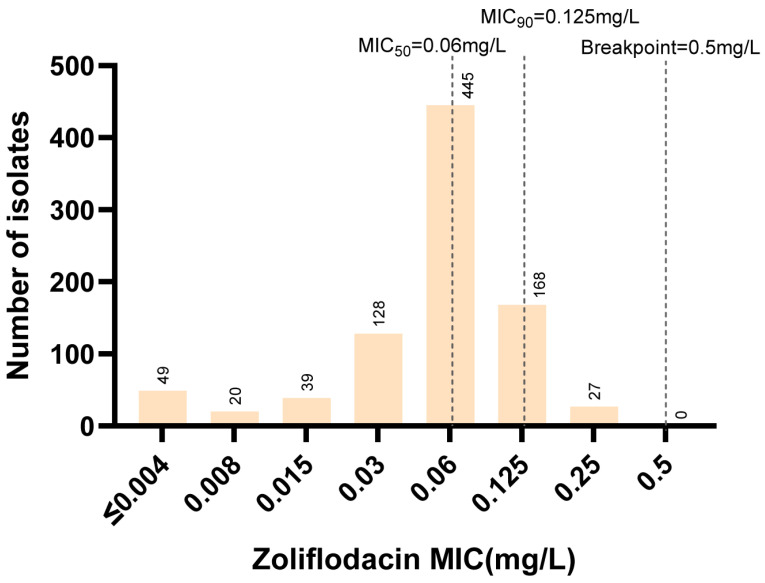
Distribution of zoliflodacin MIC of 876 isolates of *N. gonorrhoeae*.

We next determined the susceptibilities of these isolates to zoliflodacin. The overall distribution of zoliflodacin MICs ranged from ≤0.004 to 0.25 mg/L, with MIC_50_ and MIC_90_ values of 0.06 mg/L and 0.125 mg/L, respectively (Figure 1). Notably, all isolates, including those non-susceptible to ceftriaxone and azithromycin, were susceptible to zoliflodacin (Table 2), with MICs below the breakpoint of 0.5 mg/L. This indicates high in vitro activity of zoliflodacin against *N. gonorrhoeae* isolates.

**Figure 2:**
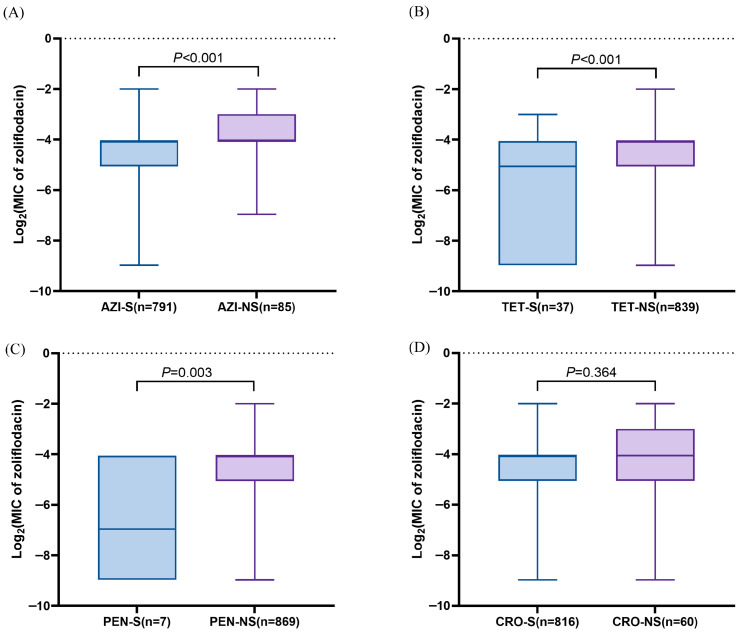
Comparisons of zoliflodacin MIC between drug-susceptible and drug-non-susceptible isolates of *N. gonorrhoeae*. (**A**) Zoliflodacin MICs in azithromycin (AZI)-S and azithromycin-NS isolates. (**B**) Zoliflodacin MICs in tetracycline (TET)-S and tetracycline-NS isolates. (**C**) Zoliflodacin MICs in penicillin (PEN)-S and penicillin-NS isolates. (**D**) Zoliflodacin MICs in ceftriaxone (CRO)-S and ceftriaxone-NS isolates. S: susceptible; NS: non-susceptible.

Comparative analysis of zoliflodacin MICs between drug-susceptible and drug-non-susceptible *N. gonorrhoeae* isolates revealed that azithromycin-non-susceptible (*p* < 0.001), tetracycline-non-susceptible (*p* < 0.001), and penicillin-non-susceptible (*p* = 0.003) isolates exhibited significantly higher zoliflodacin MICs than their susceptible counterparts. In contrast, no significant difference in zoliflodacin MICs was observed between ceftriaxone-non-susceptible and ceftriaxone-susceptible isolates (*p* = 0.364) (Figure 2). These findings suggest potential cross-tolerance between zoliflodacin and certain classes of antimicrobials, although the mechanism underlying this association remains to be elucidated.

### 2.3. Zoliflodacin Susceptibility Across Different MLST Clones

**Figure 3:**
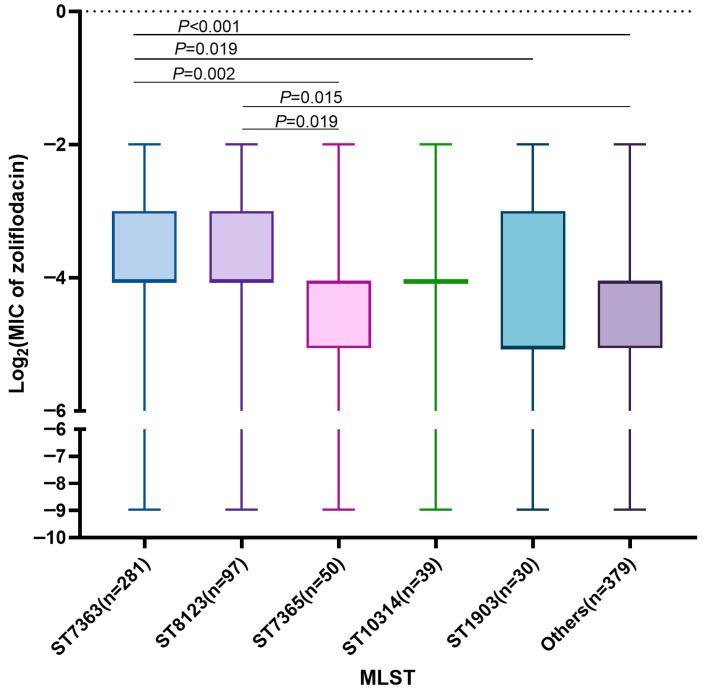
Distribution of zoliflodacin MICs across different *N. gonorrhoeae* sequence types.

Among the 876 *N. gonorrhoeae* isolates, 70 distinct sequence types (STs) were identified based on MLST. The predominant STs were ST7363 (32.08%, 281/876), ST8123 (11.07%, 97/876), and ST7365 (5.71%, 50/876), together accounting for nearly half of all isolates. Although all isolates were susceptible to zoliflodacin, their MIC values varied across different MLST clones. For example, ST7363 exhibited significantly higher zoliflodacin MICs compared with ST7365 (*p* = 0.002), ST1903 (*p* = 0.019), and the other minor sequence types (*p* < 0.001). Similarly, ST8123 showed higher MICs than ST7365 (*p* = 0.019) and the other minor sequence types (*p* = 0.015) (Figure 3). These findings suggest that certain clonal lineages may harbor genetic backgrounds conducive to increased zoliflodacin MICs.

### 2.4. Association Between Substitutions in GyrA, GyrB, ParC, ParE, and MtrR and Zoliflodacin Susceptibility in N. gonorrhoeae

Following a previous study, we investigated amino acid substitutions within the quinolone resistance-determining regions (QRDRs) of GyrA, GyrB, ParC, ParE, as well as in MtrR [15], to assess their potential impact on zoliflodacin susceptibility. In total, 8 distinct substitutions were identified in GyrA, 10 in GyrB, 17 in ParC, 7 in ParE, and 3 in MtrR (Table 3 and Table S1). The most prevalent alterations were GyrA-S91F (99.77%, 874/876), GyrA-D95A (72.95%, 639/876) and MtrR-A-53del (56.85%, 498/876). Most of the GyrB (91.10%, 798/876), and ParE (65.87%, 577/876) were wild-type.

To explore genotype-phenotype associations, isolates were categorized into lower-MIC group (MIC < MIC_90_, n = 681) and higher-MIC group (MIC ≥ MIC_90_, n = 195) based on zoliflodacin susceptibility. Univariate logistic regression analysis revealed that GyrA-D95Y (OR, 1.67; 95% CI, 1.04–2.70), ParC-D86N (OR, 1.47; 95% CI, 1.05–2.04), and MtrR-A39T (OR, 1.46; 95% CI, 1.06–2.01) were significantly associated with higher zoliflodacin MICs, suggesting a potential role in reduced susceptibility. Conversely, GyrA-D95G (OR, 0.54; 95% CI, 0.30–0.98), GyrA-D95N (OR, 0.26; 95% CI, 0.08–0.84), and ParE-P456S (OR, 0.55; 95% CI, 0.35–0.86) were correlated with lower MICs (Table 3). However, none of these associations remained statistically significant after correction, indicating that these associations may be weak.

## 3. Discussion

The increasing resistance of *N. gonorrhoeae* to first-line drugs including ceftriaxone and azithromycin underscores the urgent need for new antimicrobial agents for gonorrhea treatment [21]. Zoliflodacin, a novel spiropyrimidinetrione antibiotic, has demonstrated promising results in phase III clinical trials, showing strong efficacy against gonorrhea and offering a new therapeutic option [12]. In this study, zoliflodacin exhibits high in vitro activity against 876 *N. gonorrhoeae* isolates with varied resistance backgrounds and no resistance-associated substitutions were identified in GyrA, GyrB, ParC, ParE, and MtrR. Although overall susceptibility remained high, ST7363 and ST8123 show elevated zoliflodacin MICs compared with other sequence types. Together, these findings provide a molecular basis for zoliflodacin’s sustained antibacterial efficacy while also highlighting that resistance-associated mutations could spread clonally, emphasizing the need for ongoing monitoring as zoliflodacin moves toward clinical use.

The MIC_50_ and MIC_90_ of zoliflodacin against the 876 *N. gonorrhoeae* isolates were determined to be 0.06 mg/L and 0.125 mg/L, respectively, aligning with previous studies [13,15,17]. However, isolates with decreased susceptibility to penicillin, tetracycline, and azithromycin exhibited elevated zoliflodacin MICs compared to their susceptible counterparts. This phenomenon potentially mediated by non-target mechanisms such as enhanced efflux pump activity. For example, mutations in *mtrR* can lead to overexpression of the MtrCDE efflux pump [22], which may elevate zoliflodacin MICs in the harboring strains. However, MtrR-A39T lost significance after correction, suggesting that this substitution alone may exert only a minor effect on zoliflodacin susceptibility. Supporting this, previous studies indicated that while efflux pump overexpression can elevate zoliflodacin MICs, it is insufficient to confer full resistance in the absence of concomitant zoliflodacin-associated GyrB substitutions [20].

We identified 10 distinct GyrB substitutions, with 91.10% of isolates retaining the wild-type genotype, indicating high conservation of GyrB in clinical *N. gonorrhoeae*. Previous in vitro selection experiments under zoliflodacin pressure consistently yielded mutants with GyrB-D429N/A or K450T/N substitutions, conferring MICs of 0.5–4 mg/L [19,20,23]. Notably, these critical substitutions were absent in our clinical collection and have not been reported in other surveillance studies, suggesting they are specifically induced by zoliflodacin. Previous work by Richard A. Alm et al. demonstrated that while the GyrB-S467N substitution alone does not confer resistance, it acts synergistically with GyrB-D429N, elevating the zoliflodacin MIC to 8 mg/L [19]. A concerning route to resistance involves horizontal gene transfer; *N. macacae* has been identified as a potential source of the GyrB-S467N allele for *N. gonorrhoeae* [24,25]. The low prevalence of GyrB-S467N in our study (0.68%, 6/876) and global surveillance (0.12%) indicates that it has not disseminated via horizontal gene transfer to become a stepping-stone to high-level zoliflodacin resistance [26]. Furthermore, zoliflodacin exhibits a low spontaneous mutation frequency, and resistant mutants selected in vitro demonstrate a substantial fitness cost [19,27].

In comparison, gepotidacin, another novel oral topoisomerase II inhibitor in advanced clinical development for gonorrhea, exhibits a well-balanced dual-targeting of gyrase and topoisomerase IV [11]. Similarly to zoliflodacin, the frequency of resistance to gepotidacin is low [28]. However, a key concern is that in the context of widespread fluoroquinolone resistance, acquired substitutions such as ParC-D86N can accumulate and act as stepping-stones, enabling *N. gonorrhoeae* to develop high-level gepotidacin resistance more rapidly [29,30,31]. Therefore, while both are promising oral agents with low resistance frequencies, the different mechanisms of zoliflodacin and gepotidacin pose distinct genetic challenges and may drive divergent evolutionary paths to resistance in *N. gonorrhoeae*.

However, the emergence of resistance-associated substitutions may vary across clones. Previous studies reported that the majority of *N. gonorrhoeae* isolates carrying the GyrB-S467N substitution belong to ST7363 (74%, 37/50) [24], and the two Norwegian isolates with the highest zoliflodacin MIC (0.5 mg/L) were clonally related [32]. Our study showed that isolates belonging to ST7363 and ST8123 exhibited higher zoliflodacin MICs compared with others. ST7363 and ST8123 are reported to harbor mosaic *penA* alleles and to be associated with elevated ceftriaxone MICs [33,34,35], reflecting their capacity for clonal expansion and accumulation of resistance determinants. This suggests that once zoliflodacin-resistant-associated substitutions arise, they may be preferentially amplified within certain genomic backgrounds.

Pharmacokinetic studies have shown that a single 3-g oral dose in the single-ascending-dose (SAD) study achieved a geometric mean maximum plasma concentration (Cmax) above 20 mg/L, and mean terminal elimination half-life (t_1/2_) was 6.2 h [36]. This PK profile supports that serum concentrations can be maintained above 5 mg/L for approximately 12 h following the peak. No drug-related serious adverse events were reported (ClinicalTrials registration no. NCT03959527).

In conclusion, the high in vitro activity of zoliflodacin against *N. gonorrhoeae* observed in this study supports the potential clinical effectiveness of this novel agent. However, continued phenotypic and genomic surveillance of zoliflodacin-resistant *N. gonorrhoeae* is warranted, as mutations associated with elevated zoliflodacin MICs may emerge and spread clonally, potentially compromising its long-term clinical utility.

## 4. Materials and Methods

### 4.1. Isolation and Cultivation of N. gonorrhoeae Strains

From 2020 to 2023, a total of 876 *N. gonorrhoeae* strains were collected from patients in Shanghai. The strains were inoculated onto Thayer-Martin (TM) selective agar and cultured at 37 °C with 5% CO_2_ and controlled humidity for 18 h. After cultivation, the strains were suspended in tryptic soy broth with glycerol and stored at −80 °C for future use.

### 4.2. Antimicrobial Susceptibility Testing

MICs were determined using the agar dilution method in accordance with Clinical and Laboratory Standards Institute (CLSI) guidelines. The quality control strain used was ATCC49226. Penicillin, tetracycline, ceftriaxone, azithromycin, spectinomycin, ciprofloxacin, and zoliflodacin were purchased from MCE (MedChemExpress, Monmouth Junction, NJ, American). The breakpoint for zoliflodacin of ≥0.5 mg/L was utilized as previously described [37]. Antibiotic resistance breakpoints were applied based on the CLSI and European Committee on Antimicrobial Susceptibility Testing (EUCAST) criteria (for spectinomycin only) as follows: penicillin (MIC ≥ 2 mg/L), tetracycline (MIC ≥ 2 mg/L), ceftriaxone (MIC > 0.25 mg/L, non-susceptible), azithromycin (MIC > 1 mg/L, non-susceptible), spectinomycin (MIC ≥ 128 mg/L), and ciprofloxacin (MIC ≥ 1 mg/L) [38,39].

### 4.3. Whole-Genome Sequencing and Analysis

Genomic DNA was extracted from the 876 isolates using the bacterial genomic DNA extraction kit from TIANGEN Biotech (Beijing, China) as previous described [40]. Sequencing was performed on the Illumina HiSeq platform using 150 bp paired-end sequencing technology. Reads were assembled using SPAdes V3.8 software with default parameters, and alleles shorter than 500 nucleotides were excluded [41]. The sequence types were matched based on the pubMLST database (https://pubmlst.org/). Mutations in *gyrA*, *gyrB*, *parC*, *parE*, and *mtrR* were identified using pyngSTar with a database from CARD [42,43] and Snippy v4.6.0 (https://github.com/tseemann/snippy, accessed on 1 July 2025).

### 4.4. Statistical Analysis

The 876 isolates were categorized into a high-MIC group (MIC ≥ MIC_90_) and a low-MIC group (MIC < MIC_90_). The frequency of each genetic feature was calculated, and features with frequencies <1% or >99% were excluded. Univariate logistic regression was then performed to assess the impact of GyrA, GyrB, ParC, ParE, and MtrR substitutions on zoliflodacin susceptibility. The distributions of log_2_-transformed zoliflodacin MIC values were compared among isolates with different antimicrobial susceptibility profiles and sequence types using the Wilcoxon test. All statistical analyses were conducted using R software (version 4.3.0), with a significance level set at *p* < 0.05. *p* values were adjusted using the false discovery rate (FDR) method.

## Figures and Tables

**Table 1:** Clinical characteristics.

Characteristics	No. (%)
Sex	
Male	751 (85.73)
Female	125 (14.27)
Age	
0–19	57 (6.51)
20–39	527 (60.16)
40–59	217 (24.77)
≥60	75 (8.56)
Isolation site	
Cervix	67 (7.65)
Urethra	754 (86.07)
Vagina	55 (6.28)
Geographical region	
Urban	211 (24.09)
Suburban	665 (75.91)
Isolation year	
2020	15 (1.71)
2021	406 (46.35)
2022	264 (30.14)
2023	191 (21.80)

**Table 2:** Antimicrobial susceptibility profiles of *N. gonorrhoeae* isolates.

Antimicrobial Agents	No. of Isolates (%)			MIC (mg/L)		
	Susceptible	Intermediate	Resistant	Range	MIC_50_	MIC_90_
Penicillin	7 (0.80)	184 (21.00)	685 (78.20)	≤0.06–>16	>16	>16
Tetracycline	37 (4.22)	151 (17.24)	688 (78.54)	≤0.25–>32	8	>32
Spectinomycin	876 (100)			≤4–64	32	32
Ceftriaxone	816 (93.15)		60 (6.85)	≤0.004–1	0.03	0.25
Azithromycin	791 (90.30)		85 (9.70)	≤0.03–>4	0.25	1
Ciprofloxacin	2 (0.23)	2 (0.23)	872 (99.54)	≤0.008–>8	8	>8

**Table 3:** Substitution frequencies in clinical isolates of *N. gonorrhoeae* grouped by zoliflodacin susceptibility and univariate logistic regression analysis.

Substitution		No. of Isolates (%)			OR	95% CI	*p* Value ^1^	Adjusted *p* Value ^1^
		All Isolates (n = 876)	Isolates with MIC < MIC_90_ (n = 681)	Isolates with MIC ≥ MIC_90_ (n = 195)				
GyrA	A92P	94 (10.73)	66 (9.69)	28 (14.36)	1.56	0.97–2.51	0.065	0.175
	D95A	639 (72.95)	491 (72.10)	148 (75.90)	1.22	0.84–1.76	0.293	0.355
	D95G	99 (11.30)	85 (12.48)	14 (7.18)	0.54	0.30–0.98	0.042 *	0.161
	D95N	42 (4.79)	39 (5.73)	3 (1.54)	0.26	0.08–0.84	0.025 *	0.144
	D95Y	90 (10.27)	62 (9.10)	28 (14.36)	1.67	1.04–2.70	0.035 *	0.161
GyrB	V470I	62 (7.08)	54 (7.93)	8 (4.10)	0.50	0.23–1.06	0.071	0.175
	WT	798 (91.10)	617 (90.60)	181 (92.82)	1.34	0.74–2.45	0.339	0.390
ParC	G85C	23 (2.63)	19 (2.79)	4 (2.05)	0.73	0.25–2.17	0.571	0.625
	D86N	291 (33.22)	213 (31.28)	78 (40.00)	1.47	1.05–2.04	0.023 *	0.144
	S87C	10 (1.14)	8 (1.17)	2 (1.03)	0.87	0.18–4.14	0.863	0.902
	S87I	33 (3.77)	29 (4.26)	4 (2.05)	0.47	0.16–1.36	0.163	0.269
	S87N	106 (12.10)	77 (11.31)	29 (14.87)	1.37	0.87–2.17	0.180	0.269
	S87R	374 (42.69)	302 (44.35)	72 (36.92)	0.74	0.53–1.02	0.065	0.175
	S88P	12 (1.37)	11 (1.62)	1 (0.51)	0.31	0.04–2.45	0.269	0.344
	E91G	48 (5.48)	41 (6.02)	7 (3.59)	0.58	0.26–1.32	0.193	0.269
ParE	R427H	20 (2.28)	18 (2.64)	2 (1.03)	0.38	0.09–1.66	0.199	0.269
	D437N	112 (12.79)	80 (11.75)	32 (16.41)	1.48	0.95–2.30	0.087	0.182
	P456S	175 (19.98)	149(21.88)	26 (13.33)	0.55	0.35–0.86	0.009 **	0.144
	WT	577 (65.87)	441 (64.76)	136 (69.74)	1.25	0.89–1.77	0.196	0.269
MtrR	A39T	355 (40.53)	262 (38.47)	93 (47.69)	1.46	1.06–2.01	0.021 *	0.144
	G45D	88 (10.05)	74 (10.87)	14 (7.18)	0.63	0.35–1.15	0.134	0.257
	A-53del	498 (56.85)	398 (58.44)	100 (51.28)	0.75	0.54–1.03	0.076	0.175
	WT	14 (1.60)	11 (1.62)	3 (1.54)	0.95	0.26–3.45	0.940	0.940

^1^ Univariate regression analysis was used for the test and the substitutions with frequencies less than 1% or greater than 99% were excluded for the analysis. WT: wild-type; OR: odd ratio; CI: confidence interval; * *p* < 0.05; ** *p* < 0.01.

## Data Availability

The genome sequences of *N. gonorrhoeae* isolates have been deposited in the DDBJ/ENA/GenBank under the project of PRJNA956288.

## Supplementary Materials

The following supporting information can be downloaded at: https://www.mdpi.com/article/10.3390/antibiotics15010061/s1, Table S1: GyrA, GyrB, ParC, and ParE substitutions with frequencies less than 1% or more than 99%.
