# Supplementary material for: In Vitro Activity of Zoliflodacin Against Neisseria gonorrhoeae Isolates from Shanghai, China (2020–2023)
Source: Antibiotics (Basel). 2026 Jan 5;15(1):61. doi: 10.3390/antibiotics15010061 (PMC12837930; doi:10.3390/antibiotics15010061)
Supplement: Supplementary file 1 [file antibiotics-15-00061-s001.zip › antibiotics-4024341-supplementary.pdf]

Table S1. GyrA, GyrB, ParC, and ParE substitutions with frequencies less than 1% or more than 99%

Substitution		No. of isolates (%)		
		All isolates (n=876)	Isolates with MIC < MIC_{90} (n=681)	Isolates with MIC ≥ MIC_{90} (n=195)
**GyrA**	S91F	874 (99.77)	681 (100.0)	193 (98.97)
	D95C	4 (0.46)	4 (0.59)	0 (0.00)
	V81I	2 (0.23)	2 (0.29)	0 (0.00)
	WT	2 (0.23)	0 (0.00)	2 (1.03)
**GyrB**	L465F	2 (0.23)	0 (0)	2 (1.03)
	S467N	6 (0.68)	3 (0.44)	3 (1.54)
	Q468R	1 (0.11)	1 (0.15)	0 (0.00)
	V470L	1 (0.11)	1 (0.15)	0 (0.00)
	A471T	1 (0.11)	1 (0.15)	0 (0.00)
	A479dup	1 (0.11)	1 (0.15)	0 (0.00)
	A479V	3 (0.34)	2 (0.29)	1(0.51)
	I481M	1 (0.11)	1 (0.15)	0 (0.00)
	G482D	1 (0.11)	1 (0.15)	0 (0.00)
**ParC**	G85A	1 (0.11)	1 (0.15)	0 (0.00)
	G85D	7 (0.80)	4 (0.59)	3 (1.54)
	S87Y	1 (0.11)	1 (0.15)	0 (0.00)
	A89P	1 (0.11)	1 (0.15)	0 (0.00)
	A89T	5 (0.57)	5 (0.73)	0 (0.00)
	E91K	2 (0.23)	2 (0.29)	0 (0.00)
	E91Q	2 (0.23)	2 (0.29)	0 (0.00)
	G120E	1 (0.11)	1 (0.15)	0 (0.00)
	G120R	2 (0.23)	1 (0.15)	1 (0.51)
	WT	5 (0.57)	2 (0.29)	3 (1.54)
**ParE**	S423G	1 (0.11)	1 (0.15)	0 (0.00)
	D437H	6 (0.68)	5 (0.73)	1 (0.51)
	A443V	1 (0.11)	1 (0.15)	0 (0.00)
	L462F	2 (0.23)	2 (0.29)	0 (0.00)
